# Editorial: AI research in cancer pharmacology

**DOI:** 10.3389/fphar.2025.1681113

**Published:** 2025-10-14

**Authors:** Yookyung Christy Choi, Gregory S. Calip, Jong-Min Kim

**Affiliations:** ^1^ Global Epidemiology, AbbVie, Inc, North Chicago, IL, United States; ^2^ Department of Clinical Pharmacy, Alfred E. Mann School of Pharmacy and Pharmaceutical Sciences, University of Southern California, Los Angeles, CA, United States; ^3^ Statistics Discipline, Division of Science and Mathematics, University of Minnesota-Morris, MN, United States

**Keywords:** artificial intelligence, cancer pharmacology, drug design, pharmacovigilance, multimodal data fusion

Cancer pharmacology has traditionally employed a hypothesis-driven research paradigm. This approach typically involves testing a hypothesis, followed by a systematic investigation to derive causal inferences. While this framework provides concrete evidence and an advanced mechanistic understanding of cancer biology and pharmacological mechanisms of action, it is increasingly challenged by substantial tumor heterogeneity between types of tumors and between patient populations. Tumors evolve over time by acquiring mutations, adapting to selective pressures, and developing resistance to therapy; therefore, they exhibit temporal heterogeneity (Marusyk et al., 2010). The conventional hypothesis-first model depends heavily on prior knowledge and investigator-defined questions. Given cancer’s complex etiology and heterogeneity, this approach can create gaps in our understanding of pharmacological responses, off-target effects, resistance mechanisms, and patient-specific variability. Artificial intelligence (AI) offers a data-driven approach that can identify complex patterns within large and heterogeneous datasets. AI does not require a predefined hypothesis and is not restricted to a single data type and can integrate information from multiple sources to develop a more comprehensive understanding of pharmacological effects, utilizing high-throughput genomic sequencing, medical imaging, and electronic health records.

This Research Topic on AI in Cancer Pharmacology invited articles that applied AI computational methodologies, such as machine learning and data mining to cancer research. Based on the six featured articles, AI does not replace hypothesis-driven research; rather, it enhances the generation of empirically grounded hypotheses. All of the original studies follow a hypothesis-driven design. For example, the studies by Haq et al., Siddiqui et al., and Khalid et al. each focused on a well-characterized molecular target relevant to a specific cancer: p53 misfolding and TANK-binding kinase 1 (TBK1) in breast cancer, and platelet-derived growth factor alpha (PDGFRA) in thyroid cancer, respectively. These studies established a clear scientific rationale for the clinical relevance of each targeted biomarker and utilized multiple computational techniques, such as structure-based screening and molecular docking simulations, to identify optimal drug candidates. Taken together, the authors’ findings highlighted how AI can support a deductive research model while efficiently identifying promising drug candidates through data-driven AI approaches. The study by Siddiqui et al. was also based on hypothesis-driven research of targeting TBK1. After selecting TBK1 based on its known role in cancer, the authors applied machine learning to identify molecular features that differentiate active TBK1 inhibitors from inactive compounds. They used an Extra Trees classifier to detect complex molecular signatures, which helped prioritize compounds and supported mechanistic interpretation. This inductive step was embedded in a broader hypothesis-driven framework, illustrating how AI can expand the efficiency and scope of traditional pharmacological analyses.

Wang et al. offered a different example of AI integration, focusing on pharmacovigilance and adverse event signal detection. First, their study assessed the association between osimertinib exposure and cardiac adverse reactions (CAR) using a data-driven approach. Rather than starting with a predefined mechanistic hypothesis, the authors applied an AI-based data mining technique called Bayesian Confidence Propagation Neural Networks (BCPNN) to analyze and detect safety signals from spontaneous reports in the FDA Adverse Event Reporting System (FAERS), complementing the traditional Reporting Odds Ratio (ROR) method for signal detection. After establishing an empirical hypothesis that osimertinib is associated with CAR, the authors investigated potential mechanisms, proposing that CAR may result from multi-target interactions and pathway dysregulation. This flexibility in approach led the study to identify multi-target interactions and pathways as a plausible mechanistic explanation. How likely is it that a researcher would predefine such multi-target interactions *a priori*?

Zhang et al. provided a comprehensive review of AI-driven multimodal integration strategies in oncology, describing how different AI techniques and diverse data types, ranging from genomics and imaging to clinical notes, can be harmonized to improve cancer diagnosis, prognosis, and treatment response prediction. The authors outlined three main fusion strategies (early, intermediate, and late) for integrating different data types and discussed their applications and limitations. The review underscores the clinical potential of AI-enabled integration in enhancing biomarker discovery and patient stratification. Importantly, the authors also highlight ongoing technical and clinical challenges, such as data heterogeneity, data/model interoperability, and lack of model interpretability, while also pointing to the future role of longitudinal data and federated learning in overcoming these barriers. Rather than replacing clinical reasoning, these approaches augment it by capturing the complexity of cancer biology across data sources.

Integrating AI into cancer pharmacology research presents notable limitations. The reliability of AI outputs depends heavily on the quality, representativeness, and completeness of the input and/or reference data. All of the studies featured in this Research Topic rely extensively on *in silico* modeling based on reference databases, and each one acknowledges this limitation, expressing the need for further validation through *in vitro* or *in vivo* experimentation. This challenge is not unique to this Research Topic of articles but rather reflects a broader reality in the field. As of mid-2025, to our knowledge, no oncology therapy developed primarily through AI has received regulatory approval in the United States. While some candidates remain in clinical development, others have failed during clinical trials. A recent study estimated the phase II success rate of AI-designed drug candidates at 40%, which is in line with historical averages (Jayatunga et al., 2024), with the caveat that the sample size is small and not specific to oncology. This underscores the fact that while AI can accelerate discovery, optimize drug design, and expand our understanding of physiological effects beyond primary mechanisms of action, it cannot overcome biological complexity or replace empirical validation on its own. The emerging consensus is rather pragmatic: AI complements, but does not replace domain expertise and hypothesis-driven research (Topol, 2019; Xianyu et al., 2024). The studies in this Research Topic offer preliminary evidence of the emerging convergence of these research paradigms and point to a more integrated, adaptive, and hypothesis-informed model of biomedical discovery.
